# Genetic predisposition to tinnitus in the UK Biobank population

**DOI:** 10.1038/s41598-021-97350-z

**Published:** 2021-09-13

**Authors:** Madeleine E. Urbanek, Jian Zuo

**Affiliations:** 1 Department of Biomedical Sciences, Creighton University School of Medicine, Omaha, NE USA; 2 Department of Biology, Creighton University College of Arts and Sciences, Omaha, NE USA

**Keywords:** Midbrain, Risk factors, Genetics of the nervous system, Predictive markers

## Abstract

Tinnitus, the phantom perception of noise originating from the inner ear, has been reported by 15% of the world’s population, with many patients reporting major deficits to cognition and mood. However, both objective diagnostic tools and targeted therapeutic strategies have yet to be established. To better understand the underlying genes that may preclude tinnitus, we performed a genome-wide association study of the UK Biobank’s 49,960 whole exome sequencing participants to identify any loci strongly associated with tinnitus. We identified 17 suggestive single nucleotide polymorphisms (p < 1e−5) spanning 13 genes in two sex-separated cohorts reporting chronic, bothersome tinnitus (control males n = 7,315, tinnitus males n = 226, control females n = 11,732, tinnitus females n = 300). We also found a significant missense mutation in *WDPCP* (p = 3.959e−10) in the female cohort, a mutation which has been previously implicated in typical neuronal functioning through axonal migration and structural reinforcement, as well as in Bardet-Biedl syndrome-15, a ciliopathy. Additionally, in situ hybridization in the embryonic and P56 mouse brain demonstrated that the majority of these genes are expressed within the dorsal cochlear nucleus, the region of the brain theorized to initially induce tinnitus. Further RT-qPCR and RNAScope data also reveals this expression pattern. The results of this study indicate that predisposition to tinnitus may span across multiple genomic loci and be established by weakened neuronal circuitry and maladaptive cytoskeletal modifications within the dorsal cochlear nucleus.

## Introduction

Tinnitus, or the phantom perception of ringing in the ears, is an incredibly common condition reported by around 15% of the world’s population^1^. While it differs in severity, around 5% of those with tinnitus have particularly debilitating cases, which are associated with major cognitive deficits, including but not limited to an inability to concentrate, development of mood disorders, and sleep deprivation^2,3^. Disproportionally affecting military personnel, factory workers, and aging populations, tinnitus differs significantly in its perception and length of manifestation. Less severe, acute cases may only last for moments and only occasionally manifest in response to brief noise trauma. However, more severe, chronic cases may stretch over months and even years^4^. Despite its high prevalence, the clinical standard for diagnosis is restricted to a subjective questionnaire, and there are currently no effective therapeutic options for tinnitus^5^. Instead, the only treatment methods available are those that aim to alleviate the cognitive and mood consequences of tinnitus, rather than to diminish tinnitus itself^3^.

In order to develop effective and targeted approaches to treating tinnitus, it is critical to investigate the mechanisms underlying tinnitus’s development. A number of factors seem to contribute across a wide population: aging, particular disorders of the central nervous system, auditory dysfunction, and persistent environmental stress on these systems^6^. Immediate tinnitus onset usually occurs after severe environmental stress from intense pressure or noise, though this may differ among patients based on their individual health profiles and prior auditory damage^7^. Though the sensory cells of the inner ear may sustain damage in response to consistent environmental stress, tinnitus appears to be a neural condition, with only around 50% of tinnitus cases being directly linked to other signs of otological damage^6,8^. In particular, hyperactivity within the dorsal cochlear nucleus (DCN), a region of the brain involved in modulation of incoming auditory and cranial somatosensory signals, appears to be directly involved in the manifestation of tinnitus^9,10^.

With this neuronal circuit-based mechanism, recent research has begun to turn to differences in protein expression, and by extension, genetic inheritance as a means to explain the disparity in trauma needed for the development of tinnitus across individuals. Whether tinnitus is a heritable condition has long been debated, with mixed results indicating that tinnitus is at least slightly heritable, though likely in a complex hereditary pattern^11^. Three distinct types of genetic studies have been used to evaluate this relationship, each of which has contributed different information to supplement our understanding of tinnitus as a genetically predisposed condition. Both twin and family studies investigate the issue of heritability without need of a molecular mechanism to drive their hypothesis. These styles of studies investigate whether tinnitus is found to be inherited among individuals with higher genetic relatedness. Both types have yielded evidence that tinnitus is a heritable condition, though at relatively low to moderate rates^12–15^. Additionally, Bogo et al*.* 2017 found that while genetic influences accounted for approximately 40% of phenotypic variation in tinnitus, environment likely plays a large role in determining tinnitus onset and severity. Alternatively, Cederroth et al*.* 2019 found that adopted individuals with biological parents affected by tinnitus had a significantly higher odds ratio than those individuals without biological parents with tinnitus, implying a genetic foundation to tinnitus (OR_biological_ = 1.10 < 2.01 < 3.69 vs. OR_adoptive_ = 0.53 < 1.04 < 2.04, 95% CI). The combined results of these types of studies indicate that the predisposition to develop tinnitus may lie within an individual’s genes, but other factors likely play key roles in actually inducing the condition. Alternatively, candidate gene studies investigate genes hypothesized to be implicated in a disease’s known mechanisms. In tinnitus’ case, there have been a large number of these, the majority of which have found there to be higher expression of several nucleotide variants in genes coding for serotonin and GABA receptors, voltage-gated potassium channels, and neurotrophic factors, among others ^16–19^.

A fourth type of study used to study tinnitus includes genome-wide association studies (GWAS), unbiased screens that aim to identify single nucleotide polymorphisms (SNPs) more frequently associated with diseased patients^20–22^. Due to their specific output, the results of GWAS can also be functionally linked to known mechanisms and produce novel genes as both risk factors and therapeutic targets. The first GWAS on tinnitus failed to yield any significantly associated genes in part due to limited cohort size and inability to select for equivalent environmental trauma, and there is a clear need for a study that takes these into account^23,24^.

A more wide-scale approach is the best strategy to correctly screen for particular tinnitus subtypes while still meeting the threshold for a high-powered GWAS. Biobanks are novel databases with large amounts of biomedical information, spanning from current medical diagnoses to of exposure to environmental factors known to cause particular disorders to genomic data on participating individuals. These resources have incredible untapped potential for genetic investigations, and with their large sample numbers, they are effective in emulating population-wide genetic influences. A second study using a multi-trait analysis of hearing-related GWAS in the UK Biobank population found moderate heritability for tinnitus, as well as 20 genome-wide significant loci that overlapped with their original GWAS on hearing loss. It is likely that these loci were identified in part due to the additional power multi-trait analyses provide, and tinnitus was analyzed as a direct consequence of hearing difficulty rather than an independent condition^24^. With the UK Biobank population still unanalyzed, a third GWAS on tinnitus investigated tinnitus as an independent condition in the UK biobank and found 6 significant SNPs, with 3 of those significant SNPs also verified in the Million Veterans Program population^25^. Contrary to our study, this GWAS used genotyping array data and did not control for any noise exposure, in part because the Million Veterans Program database does not include equivalent measures. The fourth and most recent study, also in the UK Biobank population, focused on participants that reported frequent tinnitus and found three significant near the *RCOR1* gene, along with 11 other suggestive loci^26^. However, because tinnitus often manifests as a consequence of intense noise levels, a GWAS must consider the differences in environmental exposures to correctly parse out which individuals genuinely have a predisposition to tinnitus, rather than those that are simply missing the correct environmental circumstances. As the first study of WES in tinnitus, we intended to control for these circumstances and identify functionally relevant variants significantly associated in patients with tinnitus. By identifying SNPs correlated to specific amino acid changes in protein-coding regions of the genome, particular proteins with previously established functions within the brain may be implicated in tinnitus induction and subsequently serve as possible targets for further investigation. Additionally, with detailed health information on over 500,000 individuals and just under 50,000 whole exome sequences, the UK Biobank still contains novel data perfectly tailored for tackling complex conditions like tinnitus^27^.

To better illustrate the hereditary mechanisms underlying tinnitus, we performed a GWAS on these 50,000 participants to identify any suggestive or significant SNPs that were more prevalent in individuals reporting tinnitus than in those reporting no tinnitus. Because of the diversity of measures provided by UK Biobank, we were able to use a reductionist approach (in which we removed comorbidities and other confounding environmental factors while accounting for population stratification) to screen for relatively healthy participants with approximately equivalent levels of environmental exposures known to induce chronic tinnitus^28,29^. Confirmation of expression of each of the identified gene targets in the DCN of the mouse brain implicates their dysfunction as a possible developmental mechanism for the predisposition of tinnitus.

## Results

### Cohort development

**Figure 1:**
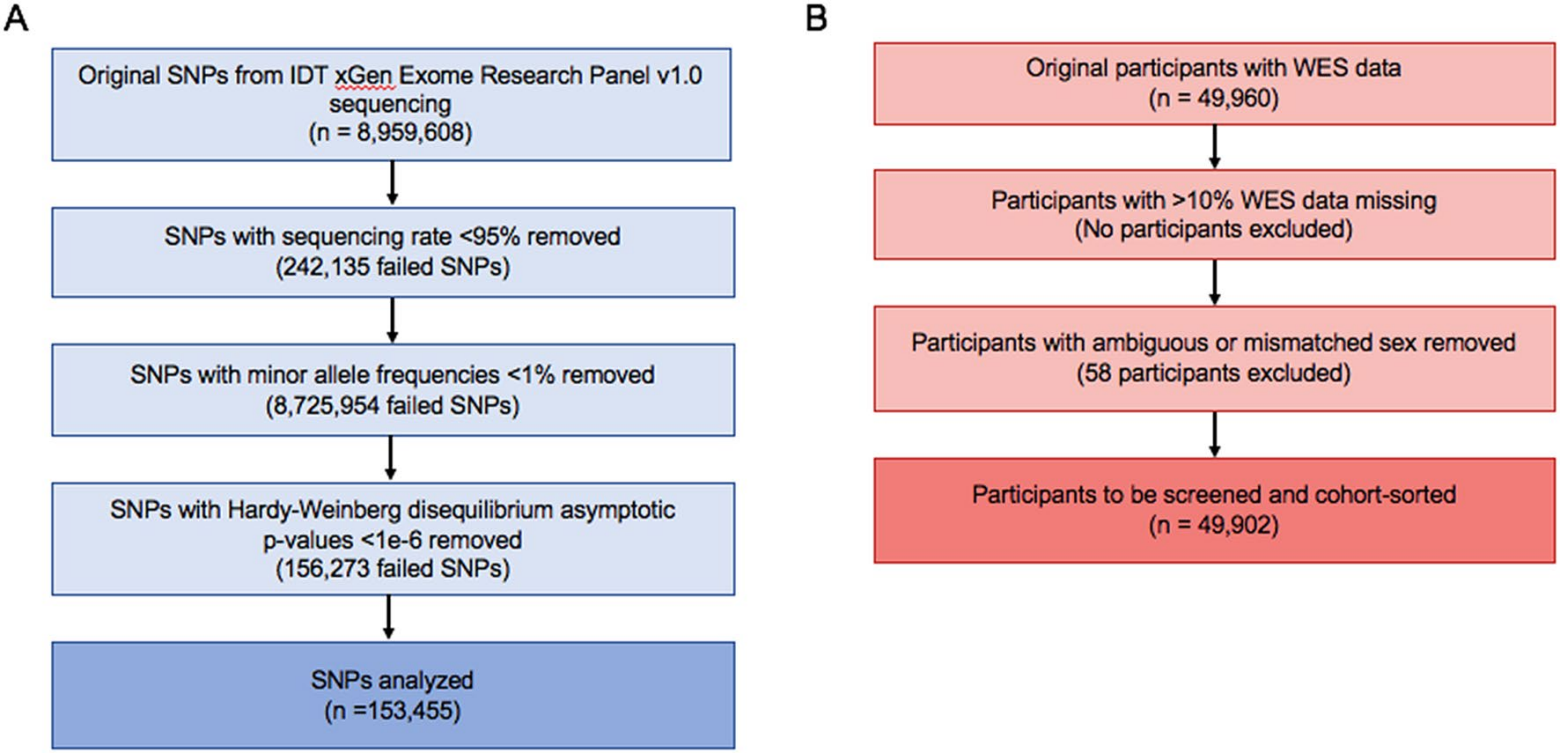
Workflow of quality control measures. Quality control measures directly applied to whole exome sequencing at the SNP (**A**) and participant level (**B**).

In order to reduce the chance of identifying rare variants that likely are not contributing to the high rates of tinnitus over more common SNPs, we ran several quality control measures to minimize the impact of these rare variants on the association analysis. Given the size of the UK Biobank’s exome-sequenced population, all thresholds for quality control were determined by the accepted values for a large population (Fig. 1)^30^. The exome sequencing panel employed by the UK Biobank originally included 8,959,608 distinct SNPs recorded across all participants. 242,135 SNPs were missing from at least 5% of the population and were removed from further analysis. Additionally, 8,725,954 of the total SNPs had a minor allele frequency of < 1% and were also removed from the analysis. Furthermore, calculated Hardy–Weinberg disequilibrium p-values < 1e−6 were found for 156,273 SNPs, and those SNPs not already eliminated from the other quality control measures were removed. This left 184,267 SNPs for the association analysis.

Of the 49,960 original participants that contributed exome sequences, all 49,960 met the threshold for having at least 90% of their sequence reported. 58 individuals had ambiguous sex reporting, due in part to missing sex-linked SNP or pedigree information and were subsequently excluded from the study. In total, 49,933 participants had adequate whole exome sequences to analyze.

Because all participants in the UK Biobank fall within a limited age range (from 40–70 years at the time of recruitment) and showed approximately equivalent distributions across this range in both tinnitus and control cohorts, we did not remove any individuals based on age (Fig S1). However, to limit the impact of genetic variation between major ethnicities, we selected individuals who were found to be genetically Caucasian by UK Biobank's genotyping methods to move forward with our study; this covered 409,605 of the total 502,492 reporting.

In order to better define our cohort, we selectively removed individuals based on official medical reporting of various diseases associated with the development of secondary tinnitus, wherein tinnitus is a side effect of a different, typically treatable condition (Table S1)^6^. To normalize the amount of environmental contribution to the development of tinnitus across the cohort, individuals with a low level of consistent noise exposure were also allowed into the study (with frequent noise exposure reduced to less than a year’s time). Controlling for noise exposure levels excludes individuals who require extremely high and frequent levels of noise exposure in order to develop tinnitus (low-threshold tinnitus) and aids in reducing the confounding effects of the environment in tinnitus’s development. Additionally, because UK Biobank reports the number of individuals recording each answer, we selected a level of noise that would be common across the population, with the vast majority of participants reporting either no extended noise exposure or less than a year’s time (98%). To account for any cochlear damage that could also be associated with heavy environmental stressors, participants were also screened for self-reported hearing loss, use of hearing aids, and use of cochlear implants (fields 2247, 3393, and 4792, respectively). These fields may better reflect phenomenon such as hidden hearing loss than a standard audiometric test and were thus utilized to ensure that hearing loss did not play a confounding role in our analysis.

These participants were then split into groups based on sex in order to account for any sex-linked genes and the sociologically driven differences in environmental trauma exposure^31–33^. To sort individuals into control and tinnitus cohorts, we used data field 4803, where the UK Biobank posed the following question to 214,040 individuals: “Do you get or have you had noises (such as ringing or buzzing) in your head that lasts for more than five minutes at a time?”. Following data-coding field 100,635, participant responses fell under three major categories, yes, no, or no answer. Any participants reporting yes also reported an approximate frequency of occurrence, from most of the time to only in the past. Because the UK Biobank collection process occurred over the course of several years, multiple assessments were performed for the majority of participants. The control cohort was comprised of those individuals reporting never having tinnitus over their multiple follow-up assessments during the UK Biobank study. Tinnitus-affected individuals were those that reported having tinnitus to some degree at least during the two most-recent follow-up reports. Though this self-report is subjective, it stands as the current clinical standard for diagnosing tinnitus. When combined with consistent reporting across multiple time points, this field serves as the best criteria for determining whether a participant has tinnitus. Furthermore, this enabled us to screen for Caucasian individuals reporting low-threshold, chronic tinnitus, which we considered to be greater than 3 months’ time.

**Figure 2:**
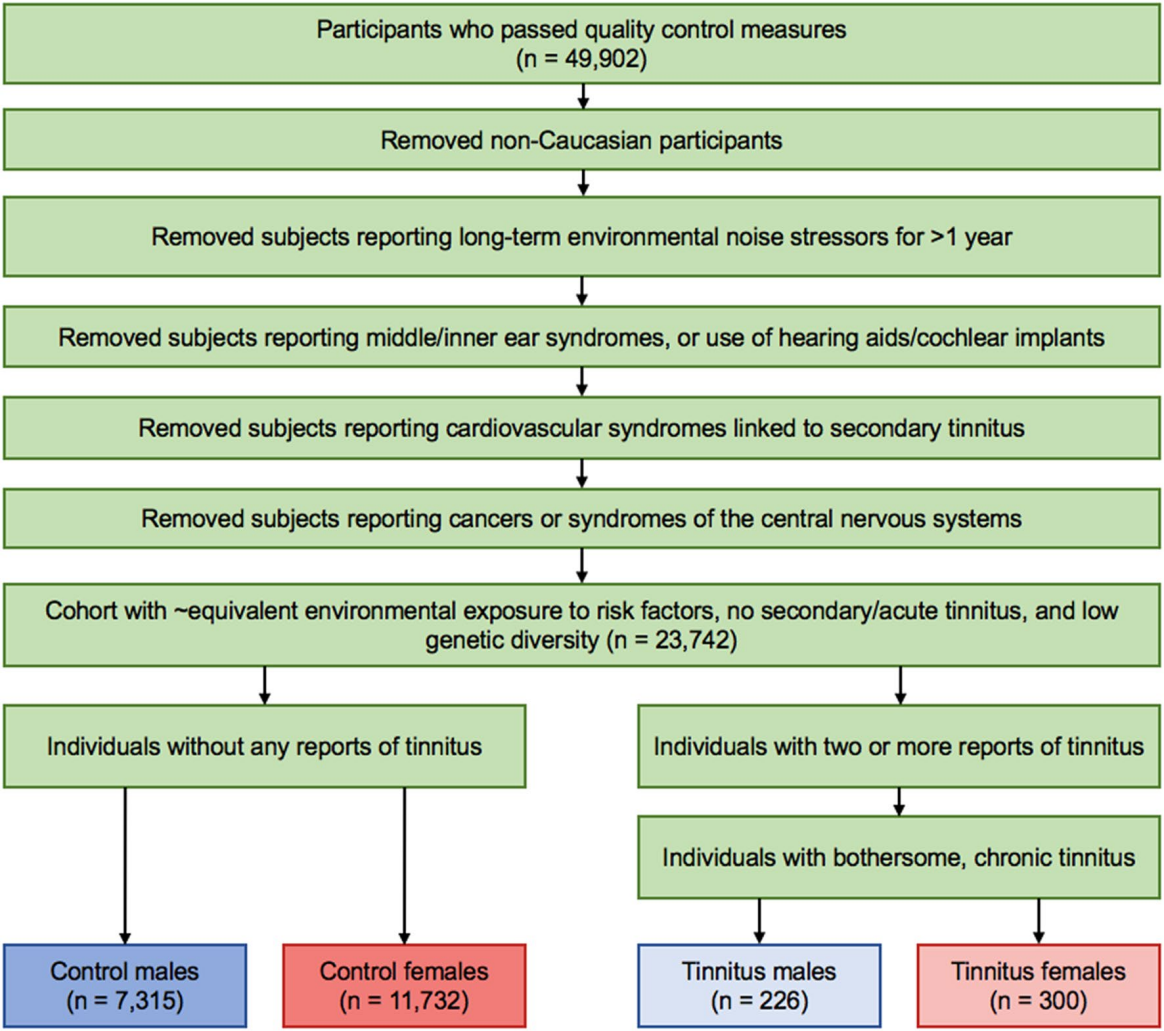
Reductionist workflow of phenotypic sorting measures used to define tinnitus and control cohorts. Participants were screened based for those with chronic, primary, low-threshold, and bothersome tinnitus against those participants never having tinnitus. Of the original 49,902 WES participants, 48,820 had self-reported tinnitus and noise stressor data.

Though the initial question posed by UK Biobank was clear and sufficient enough to screen for this subjective condition, we also wanted to further verify that our tinnitus cohort had bothersome tinnitus using the tinnitus severity field, 4814. Participants who originally reported that they had tinnitus were asked a second question, “How much do these noises worry, annoy or upset you when they are at their worst?”. Participant answers ranged from severely to not at all. We selected only participants that reported to have at least some level of bothersome tinnitus, in part to verify that these individuals were genuinely affected by the presence of tinnitus and had previously accurately reported. Individuals reporting tinnitus of no severity were excluded completely from the remainder of the study. This left 226 tinnitus cases and 7,315 controls in the male population, and 300 cases and 11,732 controls in the female population to be analyzed (Fig. 2).

### Association test

A basic association test was carried out using the plink 1.70 software, which is specifically designed to handle large cohort sizes^34^. We found a genomic inflation factor of 1.00078 in the female cohort, while the male cohort reported 1, indicating that population stratification and linkage disequilibrium were well-controlled for within our study^35^.

**Figure 3:**
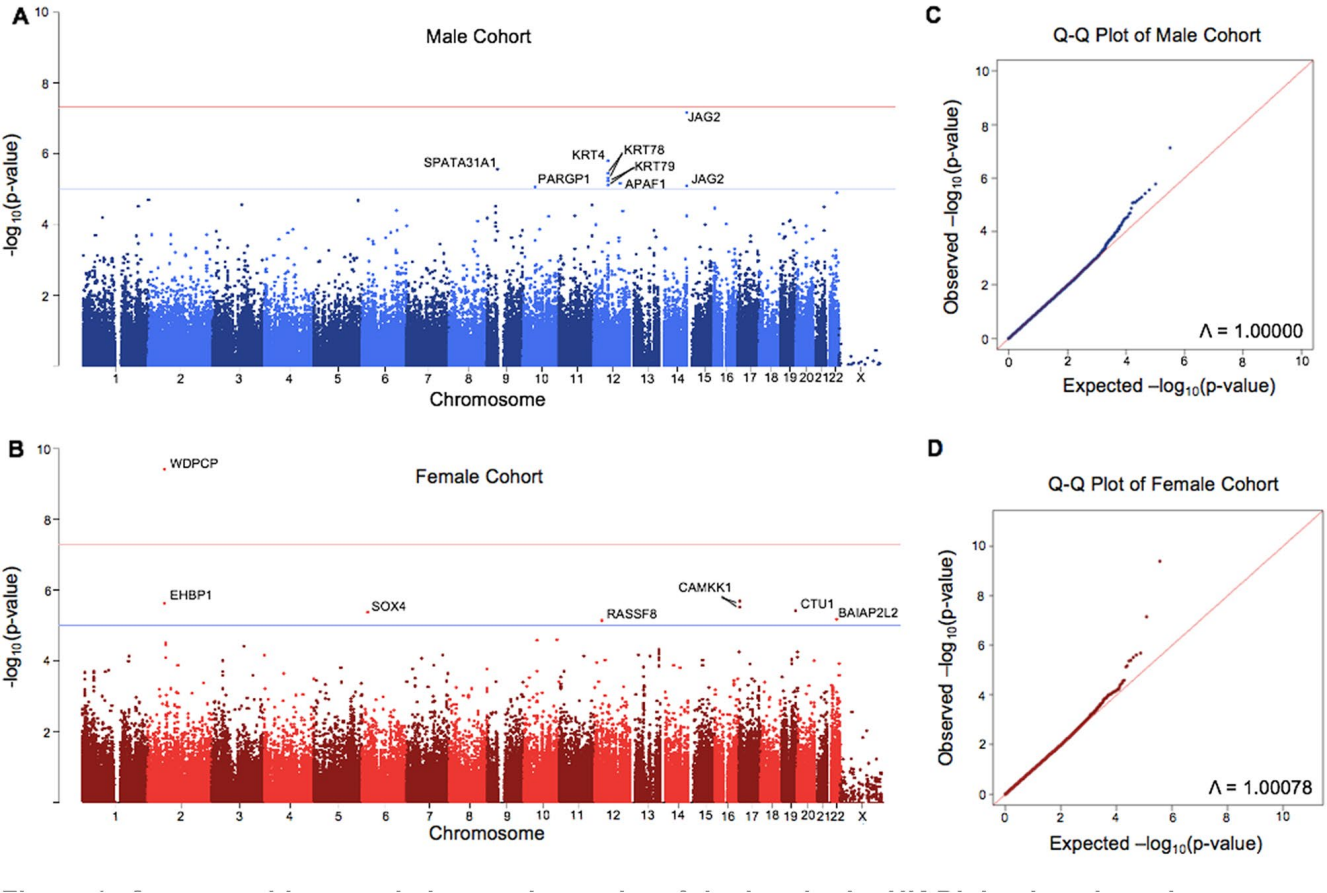
Genome-wide association study results of tinnitus in the UK Biobank male and female cohorts. The −log_10_ of each SNP’s p-values were recorded, mapped to their respective location on each chromosome, and plotted in male and female-specific Manhattan plots (**A**,**C**). Suggestive threshold is marked by the blue horizontal line at 1e−5, while the significance threshold is marked by the red horizontal line at 5e−8. In the male cohort, *JAG2*, *KRT4, SPATA31A1, KRT78, KRT79, APAF1*, *PARGP1, CAMKK1, EHBP1, CTU1, SOX4, BAIAP2L2,* and *RASSF8* were all found to be suggestively implicated in cases of chronic tinnitus, while *WDPCP* was found to be significantly implicated in the female population. Q–Q plots for each data set denoting any bias, stratification, or linkage disequilibrium are also shown (**B**,**D**). The approximate slope of 1, combined with the provided genomic inflation factors, indicates that these factors were controlled for in both populations.

The −log_10_ of the unadjusted p-values for both cohorts were recorded and mapped onto a Manhattan plot (Fig. 3). Significant and suggestive thresholds were set to the standard GWAS values, with *P* > 5e−8 and *P* > 1e−5, respectively. In the male cohort, no SNPs reached significance, while 10 fell above the suggestive threshold. In the female cohort, one SNP reached a significant threshold, while 7 met the suggestive threshold. Information on each of these SNPs was collected in Table 1. Linkage disequilibrium values (reported as R^2^) were also calculated within 1 mega base of each identified SNP. Significant and suggestive SNPs on the same chromosome were found to more frequently be associated with each other, but the majority of hits (excluding the keratin family of genes, which contained several other SNPs not identified by our analysis with R^2^ > 0.5) were found to have minimal linkage disequilibrium outside of these hits (Supplementary File 1).

**Table 1:** Identified SNPs in male (rows 1-10) and female (rows 11-18) cohorts.

SNP ID	Chromosome	Position	Gene	Mutation	Protein sequence change	Minor allele frequency	P-value	FDR BH
-	14	105,168,369	JAG2	3-base insertion	-	0.04259	7.36E−08	1.17E−02
rs17119420	12	52,810,740	KRT4	Intron variant	-	0.04748	1.66E−06	1.32E−01
rs1199015110	9	39,358,958	SPATA31A1	Missense (T > G)	Leucine to arginine	0.02691	2.84E−06	1.40E−01
rs59439901	12	52,839,305	KRT78	Silent (G > A)	-	0.04156	3.79E−06	1.40E−01
rs73106425	12	52,846,331	KRT78	Intron variant	-	0.04054	5.08E−06	1.40E−01
rs73102423	12	52,823,166	KRT79	Missense (T > C)	Lysine to arginine	0.04536	6.04E−06	1.40E−01
rs767754397	12	98,723,142	APAF1	Intron variant	-	0.01737	7.21E−06	1.40E−01
rs73106411	12	52,831,664	KRT79	Intron variant	-	0.0442	8.04E−06	1.40E−01
rs75980785	14	105,148,684	JAG2	Intron variant	-	0.03963	8.38E−06	1.40E−01
rs1287648533	10	45,953,507	PARGP1	Intron variant	-	0.01256	8.79E−06	1.40E−01
rs61734468	2	63,174,685	WDPCP	Missense (T > C)	Asparagine to serine	0.01345	3.96E−10	7.30E−05
rs74582253	17	3,882,324	CAMKK1	Missense (G > A)	Isoleucine to methionine	0.01425	2.07E−06	6.47E−03
rs78858847	2	62,859,388	EHBP1	Intron variant	-	0.01111	2.47E−06	1.12E−01
rs5610368	17	3,883,929	CAMKK1	Silent (G > A)	-	0.01257	3.05E−06	1.12E−01
rs577015585	19	51,104,412	CTU1	Missense (A > G)	Valine to alanine	0.01569	3.95E−06	1.12E−01
rs190902899	6	21,594,474	SOX4	5 prime UTR variant	-	0.01336	4.27E−06	1.12E−01
rs13058731	22	38,085,636	BAIAP2L2	Intron variant	-	0.01956	6.75E−06	1.50E−01
rs143853626	12	26,071,504	RASSF8	Intron variant	-	0.01775	7.31E−06	1.50E−01

### Analysis of SNPs and related genes

The majority of the identified SNPs were matched to their correspondent genes using the dbSNP database hosted by NCBI, which has a comprehensive list of previously identified common SNPs, their effect on the amino acid sequence, and whether they have any clinical relevance^36^. One hit, rs17119420, which coded for a 3-base insertion in *JAG2*, has not previously been recorded in dbSNP due to its identity as an insertion mutation and was instead matched to its gene using the Ensembl human genome browser^37^.

**Figure 4:**
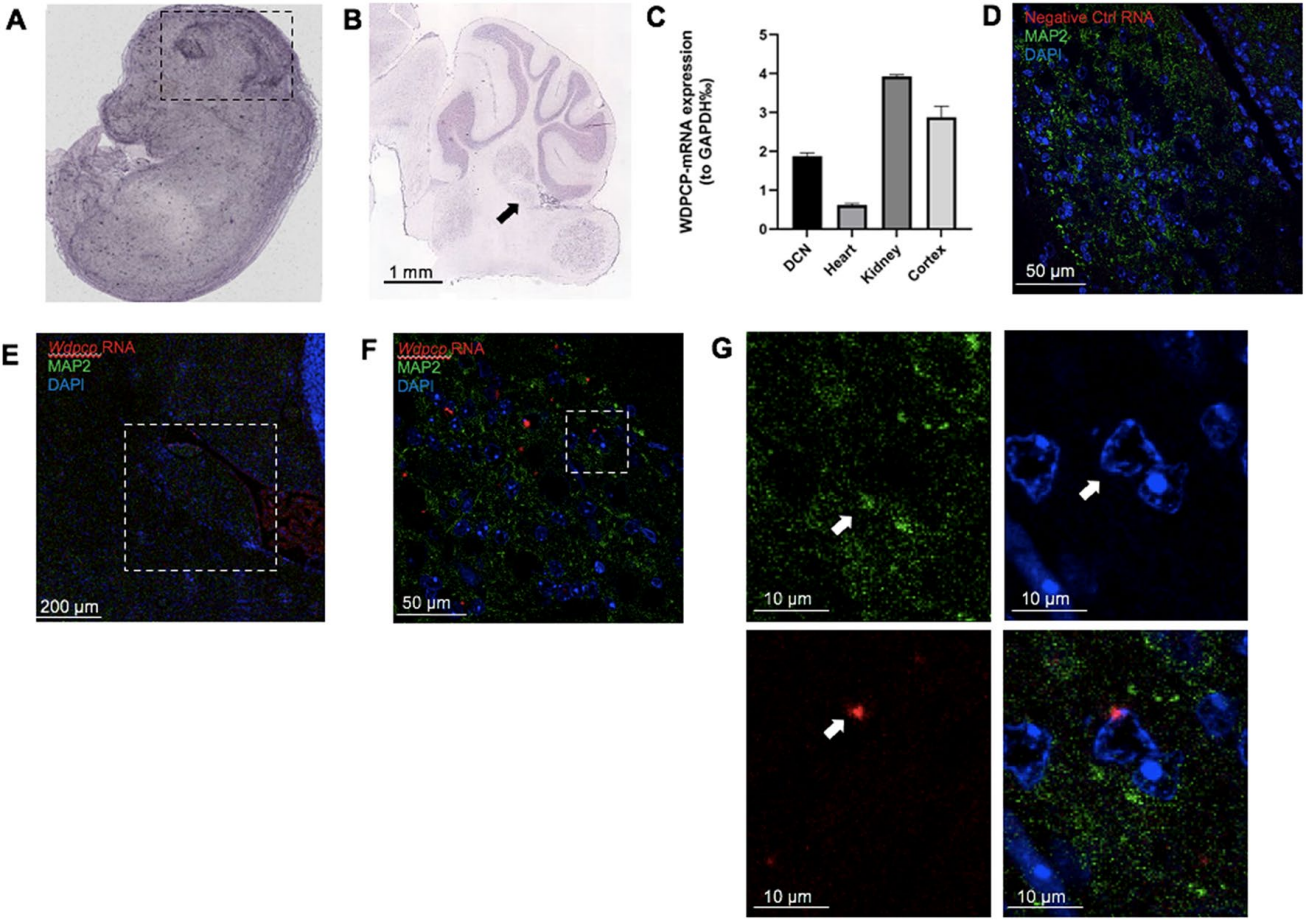
*Wdpcp* is expressed in the murine dorsal cochlear nucleus. In situ hybridization in an (**A**) 14.5 days post-conception mouse and (**B**) a C57BL/6 J mouse at P56 shows moderate expression of *Wdpcp* within the brain and DCN compared to surrounding tissues. The embryonic brain is demarcated by black dotted lines, while the DCN in the adult in situ data is marked by a black arrow. Quantification of *Wdpcp* RNA expression in a 2-month-old mouse shows relatively equivalent amounts in both the dorsal cochlear nucleus and cortex (**C**). Immunofluorescent imaging in 3-month-old mice combined with RNAScope shows colocalization of *Wdpcp* RNA in neurons (n = 5). Negative controls treated with a zebrafish *slc17a8* probe at 40 × magnification (**D**). The DCN is outlined in white in the 10X panel (**E**). Images at 63 × show distinct *Wdpcp* RNA puncta (**F**). Enlargements of the space enclosed in the white box in panel F show colocalization of *Wdpcp* RNA in nuclei surrounded by MAP2 (**G**). White arrows denote location of MAP2 expression, nucleus of interest, and *Wdpcp* RNA puncta in split channels. Image credit: EMAGE Project (EMAGE: 18,852), and Allen Institute (https://mouse.brain-map.org/experiment/show?id=69012956).

With each SNP now matched to a gene, we investigated whether these genes were expressed within both the embryonic mouse brain and the adult DCN using the EMAGE database and Allen Brain Atlas, respectively. The EMAGE database makes use of whole-mount in situ hybridization data 3-dimensionally mapped to mouse embryos 14.5 dpc and includes original data from the EurExpress project^38,39^. This in situ data is sourced from literature, screening projects, and direct submissions where it is then processed by the EMAGE consortium. Within the EMAGE database, there is clear expression of *Wdpcp* within the brain, as well as several of our suggestive SNPs (Fig. 4A, Fig S2). The Allen Brain Atlas uses in situ hybridization using horseradish peroxidase-linked riboprobes in P56, C57BL/6 J mice that mark gene expression across multiple planes of the brain^40^. Riboprobes were used for all mRNA-specific staining and run in parallel with antisense *Drd1a* and non-probe samples to verify signal quality, intensity, and specificity, in accordance with the quality control standards set by the Allen Brain Atlas^41^. The protocol was designed to minimize non-specific binding of the riboprobe and control for any background hybridization, and all controls were verified by Allen personnel prior to uploading the riboprobe images to the database. Within the database, we found mRNA expression for all identified genes in the DCN in adult mice, indicating that each of these genes may play some role in regulating cell survival and function in the adult rodent brain, and thus contribute to tinnitus (Fig. 4B, Fig S3). Furthermore, *Wdpcp* mRNA expression was measured using RT-qPCR in a 2-month-old C57BL/6 J mouse, which demonstrated moderate expression within the DCN as compared to control heart and kidney tissues, both of which have been reported to express *Wdpcp* (Fig. 4C)^42^. Despite multiple attempts to use commercially available antibodies for WDPCP to measure protein expression, these antibodies failed in both western blot and immunostaining (sc-514151, Santa Cruz Biotechnology, NBP1-92,580, Novus Biologicals, and ABIN6826045, Antibodies Online). As an alternative, we generated RNAScope imaging data from 1-month-old C57BL/6 J mice coupled with immunofluorescent labeling of neurons to determine whether *Wdpcp* is expressed within neurons of the DCN^43^. While the *Wdpcp* signal was fairly moderate, colocalization could be seen with specific neurons within the DCN using MAP2 as compared to negative controls (Fig. 4D–G). Additional staining with TUJ1 antibodies yielded similar results, as well as in cryosectioned tissue (data not shown).

We also conducted a literature search on our top hit gene, the WD Repeat Containing Planar Cell Polarity Effector *WDPCP*, in order to determine whether it had been previously associated with other dysfunctions of the central nervous system. WDPCP uses PCP effector proteins to regulate ciliogenesis during development, and it appears to have downstream consequences on regulation of the actin cytoskeleton^42^. While no tinnitus studies have investigated or found WDPCP as a potential target, our significant SNP has been identified as a driver in the development of Bardet-Biedl syndrome-15, an autosomal recessive disorder that results in photoreceptor degeneration in the retina, cognitive deficits, and delayed hearing loss^44^. Additionally, *Wdpcp* mutant mice were shown to have abnormal hair cell patterning within the inner ear, potentially due to disruptions to signaling cascades during embryonic development^42^.

## Discussion

Through our GWAS, we identified 17 suggestive and 1 significant SNP that were found to be more highly expressed in a tinnitus-afflicted population. Due to the extensive information measures recorded by the UK Biobank database, we were able to selectively screen individuals that suffer from chronic, bothersome, low-threshold tinnitus in a relatively healthy, Caucasian population. Additionally, the sheer size of the UK Biobank’s whole exome sequencing population allowed us to apply stringent phenotypic sorting measures to carefully define both cohorts and minimize the effect of environmental differences across cohorts. Because of the increased power of our GWAS granted by our large cohort sizes, we were also able to use sex-separated cohorts to further control for any environmental differences typically experienced disproportionately between sexes, and well as to identify sex-specific genes. Though there was no clear overlap between our cohort results, this is expected due to tinnitus’s multigenic inheritance pattern, which results in numerous SNPs contributing “missing heritability” to the development of tinnitus, with their expression differing from population to population^45^. This same pattern was seen when analyzing the remainder of the UK Biobank population that reported heavy noise exposure; there was minimal overlap in suggestive SNPs, and we also found no significant SNPs associated with this cohort.

Though our GWAS is not the first to be conducted on the participants in the UK Biobank cohort, we made use of a novel set of data, the whole exome sequences, which could lead to completely distinct loci from those previously found^25,26^. Because the majority of our data spanned protein-coding and adjacent regions of the genome, six of our SNPs resulted in direct alterations to the amino acid sequence. These SNPs provide straight-forward opportunities to study the direct consequences of these changes both in vitro and in vivo, which may prove critical in further elucidating the underlying pathology predisposing individuals to tinnitus. Our carefully collated cohort, while reducing the total number of participants and subsequent power of our GWAS, allowed us to analyze a specific sub-type of chronic, bothersome tinnitus, while ensuring that our cohorts had received approximately equivalent levels of noise exposure. Because tinnitus is so closely connected to environmental traumas, it is critical that further GWAS into tinnitus take this into account in order to minimize false cohort sorting^46^. This was reflected in our results, as even with the minor reduction in power combined with the normally disperse effects of tinnitus’s low heritability, we were still able to determine a number of suggestive SNPs, along with our top hit, *WDPCP*. Additionally, this approach to phenotypic cohort sorting, along with tinnitus’s complex heritability pattern, may also serve to explain why our cohort found no overlaps with those of other tinnitus GWAS^23,25,26^. Even GWAS investigating larger populations of more general tinnitus lack overlap with one another, indicating that further characterization of identified SNPs and their influence on varying tinnitus phenotypes is needed.

Of our suggestive hits, several of the genes have been found to play critical roles in cytoskeletal support, as well as in developmental and calcium-based signaling. For example, the keratin family of genes code for intermediate filament proteins, most commonly found in epithelial tissue. While the roles of *KRT78* and *KRT79* haven’t been fully characterized within the brain, the SNP rs89962 has been founded to be associated with increased risk of stroke^47^. Another suggestive hit, *BAIAP2L2* (a stereociliary protein found in both inner and outer hair cells) has been shown to have profound effects on hearing loss in mutant mice^48^. While these hits indicate that cellular structure may play a role in predisposing individuals to tinnitus, signaling proteins may also play much more complicated roles that warrant further investigation. For example, the JAG2 protein has been found to be expressed in nearly all postnatal neurons in mice, hinting at its critical role in Notch-DSL signaling^49^. The scope of the effects of each of the SNPs identified in this study, however, remain to be further investigated.

Our significant SNP in the gene *WDPCP* has also been previously implicated in a disease with major neurological degeneration, Bardet-Biedl syndrome-15, indicating that this mutation may have long-term consequences on the survival and function of affected neurons^50^. Though Bardet-Biedl syndrome-15 is often linked to improper development of sensory hair cells, leading to eventual hearing loss, our GWAS eliminated participants that had previously reported hearing loss, the use of cochlear implants, or hearing aids, indicating that our results were not due to an oversaturation of BBS-15 patients within our tinnitus group. Additionally, BBS-15 has a prevalence of 1:100,000–160,000 in North American and European populations respectively, further indicating that it was not a major contributor to our tinnitus phenotype^51^. As for the case of tinnitus, our particular *WDPCP* SNP is an asparagine to serine missense mutation, which may impede or alter the known functions of *WDPCP*. Because our final population lived to an elderly age and had no reports of major CNS conditions, we hypothesize that our *WDPCP* mutation may first cause malfunctions during embryonic development that are not severe enough to fully disrupt maintenance of cytoskeletal structure in neurons after this period. Forming the core structure of the ciliogenesis and planar polarity effector complex, WDPCP is directly involved in ciliary communication necessary for coordinating changes in the cytoskeleton for proper axonal migration^52^. Additionally, primary cilia also respond to changes in Sonic hedgehog and Wnt signaling to initiate transduction pathways altering gene expression^53^. Mutations in 187 genes related to ciliogenesis, also known as ciliopathies, have currently been linked to 35 different ciliopathies, with an additional 241 related genes likely to be involved in the ciliopathies^54^. Given that primary cilia function is critical for the proper development of the CNS, it is no surprise that many of these ciliopathies result in major neurological defects, including neural malformation, intellectual disabilities, and ataxia^55^.

We hypothesize that post-development, alterations in ciliogenesis and modulation of the septin-linked actin cytoskeleton result in deficient formation and maintenance of neuronal circuits within the DCN. Within the DCN, fusiform cells are responsible for integrating multi-sensory information (including somatosensory inputs from the trigeminal ganglion, spinal trigeminal nucleus, cervical dorsal root ganglion, and dorsal column nucleus with auditory input from the auditory nerve fiber) and subsequently relaying this information further through the auditory pathway. In part due to their modulatory nature, these fusiform cells are highly adaptive and undergo synaptic plasticity as a core mechanism to integrate multiple incoming signals. NMDA-mediated Hebbian plasticity in fusiform cells may result in post-synaptic long-term potentiation, increasing the response of fusiform cells to incoming signals^56,57^. As for molecular mechanisms after tinnitus induction, there appears to be restructuring of NMDA receptors decreasing the spontaneous activity of the inhibitory cells of the DCN^9^. This shift in excitation/inhibition within the DCN could likely play a role in subsequent fusiform hyperactivity. Additionally, structural consequences of plasticity within the DCN point to abnormal axonal sprouting and redistribution of glutamatergic projections. Upregulated expression of VGLUT2 has implicated increased excitatory signaling along somatosensory projections, while downregulated expression of VGLUT1 along the auditory nerve may result in tonic activation and subsequent hyperactivity^58^. These changes have also been found to be coupled with reductions in auditory projections and mirrored increases in non-auditory projections, which may further disrupt the carefully balanced circuitry of the DCN^59^. Restructuring of synapses and neuronal circuitry is a common theme posited in each of these mechanisms; subsequently, *WDPCP*’s role in facilitating these structural changes could underscore tinnitus’s identity as a misdirected plasticity response within the DCN.

In conclusion, our study provides evidence that individuals may be predisposed to tinnitus through multiple mutations across several genes likely contributing to its development. The identification of our significant SNP in *WDPCP* suggests a possible molecular mechanism underlying the previously established DCN hyperactivity hypothesis, which will require further investigation to properly identify the exact role *WDPCP* mutations may play. Additionally, it appears that, while environmental trauma may be needed to instigate tinnitus, inherited mutations in a combination of our identified genes may provide the developmental framework for flawed neuron structure and synapses that predispose an individual to tinnitus. Further investigations into tinnitus, should continue to focus on carefully defining the tinnitus phenotype in large populations in order to continue parsing out this developmental framework, as well as any other factors which may contribute to tinnitus’s development. With its plan to continue releasing additional sets of whole exome sequences, the UK Biobank still holds potential to both verify our findings and uncover additional SNPs with the increased power provided by a larger cohort. Further expansion in additional populations, especially those of different ethnicities and geographical regions, will also prove critical for understanding the genetic foundation of tinnitus. Additionally, a study into the molecular mechanisms supported by the findings of any future GWAS may prove useful for improving our pathological knowledge of tinnitus.

## Methods

### Data retrieval from UK biobank

Whole exome sequences were retrieved from 49,960 individuals by Regeneron Genetics Center collection team using a modified version of the IDT xGen Exome Sequencing Research Panel v1.0. These sequences were downloaded from UK Biobank’s servers in an encrypted format, along with demographic and data and those fields related to hearing, central nervous diseases, and cardiovascular diseases implicated in secondary tinnitus. All participants provided written, informed consent to participate, with consent maintained by the parent studies. This study was formally approved by the UK Biobank (Project ID 57,266), and all data were analyzed anonymously in accordance with the guidelines and regulations established by the UK Biobank.

### Quality control measures

All quality control measures and association analyses were executed through the plink v1.07 software package^34^. All SNPs were first filtered to exclude those with an exome-wide sequencing rate of less than 95% using the –geno 0.05 command. Those SNPs with a minor allele frequency of less than 1% were removed using the –maf 0.01 command, and any SNPs with Hardy–Weinberg disequilibrium p-values < 1e−6 excluded using the –hwe2 0.000001 command.

Individuals with missing sequencing data of > 10% were removed using the –mind 0.10 command. Additionally, all participants with ambiguous sex codes were automatically disqualified from the study and defined as missing in the .fam file.

### Phenotypic identification and cohort sorting

Individuals with clinically diagnosed medical conditions known to induce secondary tinnitus (selected from hearing, central nervous system, and cardiovascular disorders) were removed from the study via manual input into the .fam file. Each report was referenced to the UK Biobank’s data-coding field 2171, which described the source of diagnosis. Death registers, reports by primary care physicians, and hospital admissions were all considered to be valid diagnoses, and a report of any of those in the participant’s history resulted in total exclusion of that participant from the study. Participants self-reporting loud workplace or music exposure for greater than a year’s worth of consistent exposure were removed. Additionally, participants reporting total deafness or the use of auditory devices indicative of major hearing loss, such as cochlear implants or hearing aids, were excluded.

Cohort sorting into either the control or tinnitus phenotype was based on the data available from UK Biobank’s reported tinnitus field, 4803. Presence of tinnitus in the tinnitus cohort was verified using the tinnitus severity field, 4814. Any individuals reporting any level of bothersome tinnitus were included in the tinnitus-affected cohort, while those individuals reporting either no tinnitus severity or answer were manually excluded from the study using the .fam file.

### Association analysis

Both cohorts were divided into male and female sub-cohorts and run individually based on genetic sex provided in field 31. Each cohort was analyzed using the–assoc command, which performs a basic association analysis to compare allele frequencies between the tinnitus-affected and control individuals. Additionally, the –adjust command was used to correct for each significance test automatically executed by the –assoc command. Chromosome number, SNP ID, unadjusted p-values, and FDR values were reported for all SNPs analyzed. Significance threshold was set at *P* < 5e−8, and suggestive threshold was set at *P* < 1e−5.

### SNP and gene identification

Suggestive and significant SNPs were individually run through NCBI’s dbSNP database to identify their gene, mutation type, and previously reported clinical relevance. The *JAG2* hit, which hasn’t been recorded in dbSNP due to its identity as an insert mutation, was instead matched to the human genome using the Ensembl database.

Presence of the mRNA of identified gene was matched to the Allen Brain Atlas using sagittal in situ hybridization samples from C57BL/J6 P56 male mice as detailed in their protocol documentation.

Additionally, embryonic mRNA expression was evaluated using the eMouseAtlas (EMAGE gene expression database (http://www.emouseatlas.org/emage/), which contains whole-body in situ hybridization images in mice 14.5 dpc [].

### RT-qPCR

Procedures used with mice were approved by the Institutional Animal Care and Use Committee (IACUC) at the Creighton University. Fresh tissue cortex, primarily the DCN, heart, and kidney were dissected from a C57BL/6 2-month-old mouse. RNA was extracted using RNAqueous total RNA Isolation Kit (Life Technologies) according to manufacturer's instructions. Total RNA was then converted to cDNA using the iScript gDNA Clear cDNA Synthesis Kit (BIO-RAD Cat.#1,725,035). RT-PCR was performed using PowerUp SYBR Green Master mix (A25741). Each sample was run in triplicate along with the housekeeping gene, GAPDH. Relative quantities of the transcripts were determined using the ΔCt method using GAPDH as a reference. Standard dose–response curves of the primers yielded a slope of −3.2 to −3.0. Primer sequence: WDPCP Forward: CAGCAACAGAGCATGGTAAC, WDPCP Reverse: AGTTCCTCCAACTCCTTCAG.

### RNAScope

Expression of *Wdpcp* was examined in 1-month-old male and female mice (n = 5). Mice were transcardially perfused with 4% PFA and brains were dissected out and embedded in either paraffin or OCT. The brains were sectioned in 10 μm slices as per the RNAScope protocol. After sectioning, brain slices were mounted on SuperFrost Plus slides and baked at 60 °C for 1 h using the HybEZ Oven. Tissues were then deparaffinized in xylene twice for 5 min, then 100% ethanol twice for 1 min. Using an RNAScope 2.5 HD Reagent Kit-RED (#322,350, ACDBio), each tissue was covered in RNAScope hydrogen peroxide solution for 10 min, then washed twice in distilled water. Sections were then incubated with Protease Plus for 10 min at 40 °C, then washed in distilled water. Mouse *Wdpcp* (#502,151, ACDBio) and the control probe *slc17a8* in zebrafish (#549,991, ACDBio) were then hybridized for 2 h at 40 °C. Sections were washed twice for 2 min at RT in 1X wash buffer. AMPs were hybridized for either 15 or 30 min at 40 °C or RT, as detailed by the RNAScope 2.5 HD Protocol. After each AMP was hybridized, slides were washed twice for 2 min with RT 1 × wash buffer. Following amplification steps, sections were treated with a 1:60 ratio of Fast RED-B to Fast RED-A for 10 min in the dark at RT. Slides were then washed with distilled water twice.

### Immunofluorescence

RNAScope was combined with immunofluorescence to confirm colocalization of *Wdpcp* within specific neurons of the DCN. Immediately after completing the RNAScope protocol, slides were washed twice with 1X PBS for 2 min each, then once for ten minutes. The slides were then incubated with PBS/Triton 0.25% twice for ten minutes. For blocking, sections were incubated in the dark at RT with a 5% goat serum solution in PBS/Triton 0.25% for one hour. After incubation, slides were treated with a 1:200 dilution of MAP2 antibody (MAB3418, Millipore Sigma) or 1:1000 TUJ1 antibody (MMS-435P, Covance) in 5% goat serum/PBS/Triton 0.25% and left to incubate overnight at 4 °C. The following day, slides were washed twice with PBS for 10 min, then PBS/Triton 0.25% for 10 min. For fluorescent staining, slides were treated with DAPI (DAPI, Thermo Fisher, 1:1000) and donkey anti-mouse IgG H + L AF-488 (#A-21202, ThermoFisher, 1:1000) or goat anti-mouse IgG2a AF-647 (#A-21241, ThermoFisher, 1:1000) for 1 h in the dark at RT. Slides were then washed three times with PBS for 10 min each. Sections were then cover-slipped using Fluoromount-G medium (SouthernBiotech, USA). Slides were imaged using a confocal microscope (Zeiss LSM 710) at 10 × and oil immersion objective 63x. Images were processed using Imaris software (Biplane, USA). Processing parameters were maintained across samples.

## Supplementary Information

Supplementary Information 1.

Supplementary Information 2.
