# Supplementary material for: Genetic predisposition to tinnitus in the UK Biobank population
Source: Sci Rep. 2021 Sep 13;11:18150. doi: 10.1038/s41598-021-97350-z (PMC8437971; doi:10.1038/s41598-021-97350-z)
Supplement: Supplementary file 2 — Supplementary Information 2. [file 41598_2021_97350_MOESM2_ESM.pdf]

Genetic predisposition to tinnitus in the UK Biobank population

Madeleine E Urbanek[1,2], Jian Zuo[1*]

1) Department of Biomedical Sciences, Creighton University School of Medicine, Omaha, Nebraska, United States of America

2) Department of Biology, Creighton University College of Arts and Sciences, Omaha, Nebraska, United States of America

*jianzuo@creighton.edu

Supplementary Information

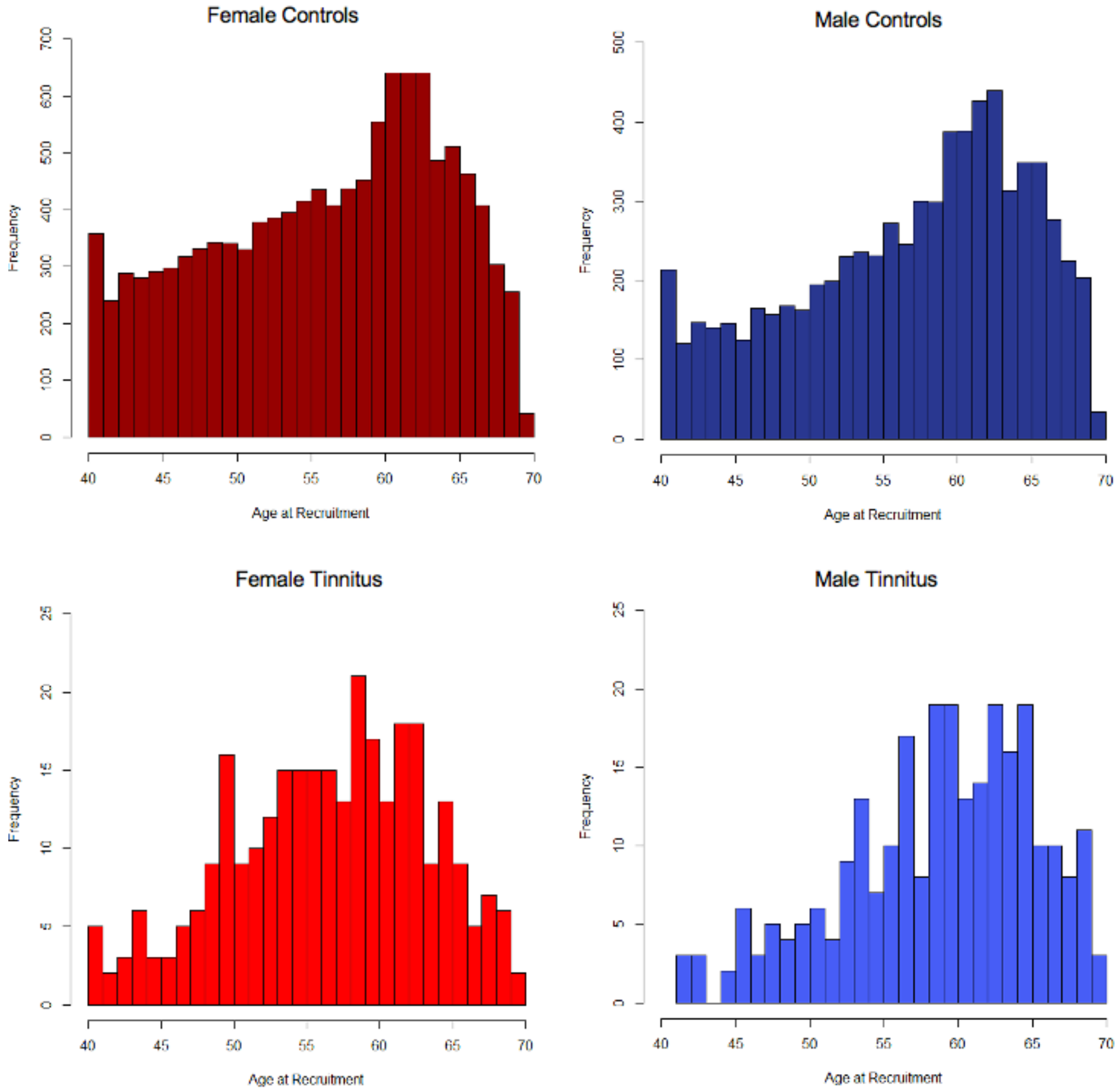

Fig S1. Age distribution of all included participants in both tinnitus and control cohorts. At the time of recruitment, all participants fell between 40 and 70 years of age. In all four groups, distribution was approximately equivalent, with the peak of participants being 60-65 years old.

	Field Subject	Field Number	Categories Excluded
**Ethnicity**			
	Genetic ethnic grouping	22006	Non-Caucasian
**Environment**			
	Noisy workplace	4825	Yes for ≥ 1 year
	Loud music exposure	4836	Yes for ≥ 1 year
**Hearing**			
	Hearing difficulty	2247	Yes or total deafness
	Hearing aid	3393	Yes
	Cochlear implant	4792	Yes
	Unsuppurative otitis media	131229	Death, hospital admissions, primary care records
	Suppurative otitis media	131231	Death, hospital admissions, primary care records
	Eustachian obstruction	131235	Death, hospital admissions, primary care records
	Mastoiditis	131239	Death, hospital admissions, primary care records
	Choleseatoma of middle ear	131241	Death, hospital admissions, primary care records
	Perforation of tympanic membrane	131243	Death, hospital admissions, primary care records
	Otosclerosis	131251	Death, hospital admissions, primary care records
	Conductive and sensorineural hearing loss	131259	Death, hospital admissions, primary care records
	Otalgia and effusion of ear	131263	Death, hospital admissions, primary care records
**Cardiovascular**			
	Stroke	42006	Death, hospital admissions, primary care records
	Hypertensive disease	131289	Death, hospital admissions, primary care records
**Central Nervous System**			
	Type of cancer	40006	Malignant neoplasms of eye, brain, and other parts of CNS
	Bacterial meningitis	130993, 130995	Death, hospital admissions, primary care records
	Meningitis	130997, 130999	Death, hospital admissions, primary care records
	Encephalitis, myelitis, and encephalomyelitis	131001, 131003	Death, hospital admissions, primary care records
	Intracranial and intraspinal abscess and granuloma	131005, 131007	Death, hospital admissions, primary care records
	Intracranial and intraspinal phlebitis and thrombophlebitis	131009	Death, hospital admissions, primary care records
	Huntington's disease	131013	Death, hospital admissions, primary care records
	Hereditary ataxia	131015	Death, hospital admissions, primary care records
	Spinal muscular atrophy	131017	Death, hospital admissions, primary care records
	Postpolio syndrome	131021	Death, hospital admissions, primary care records
	Parkinson's disease	131023	Death, hospital admissions, primary care records

	Secondary Parkinsonism	131025	Death, hospital admissions, primary care records
	Dystonia	131031	Death, hospital admissions, primary care records
	Alzheimer's disease	131037	Death, hospital admissions, primary care records
	Multiple sclerosis	131043	Death, hospital admissions, primary care records
	Epilepsy	131049	Death, hospital admissions, primary care records
	Status epilepticus	131051	Death, hospital admissions, primary care records
	Transient cerebral ischaemic attacks	131057	Death, hospital admissions, primary care records
	Vascular syndromes of the brain in verebrovascular disease	131059	Death, hospital admissions, primary care records
	Disorders of the trigeminal nerve	131063	Death, hospital admissions, primary care records
	Facial nerve disorders	131065	Death, hospital admissions, primary care records
	Nerve root and plexus disorders	131071	Death, hospital admissions, primary care records
	Myasthenia gravis and other myoneural disorders	131093	Death, hospital admissions, primary care records
	Infantile cerebral palsy	131101	Death, hospital admissions, primary care records
	Hydrocephalus	131111	Death, hospital admissions, primary care records
	Toxic encephalopathy	131113	Death, hospital admissions, primary care records

Table S1. UK Biobank fields disqualified from GWAS.

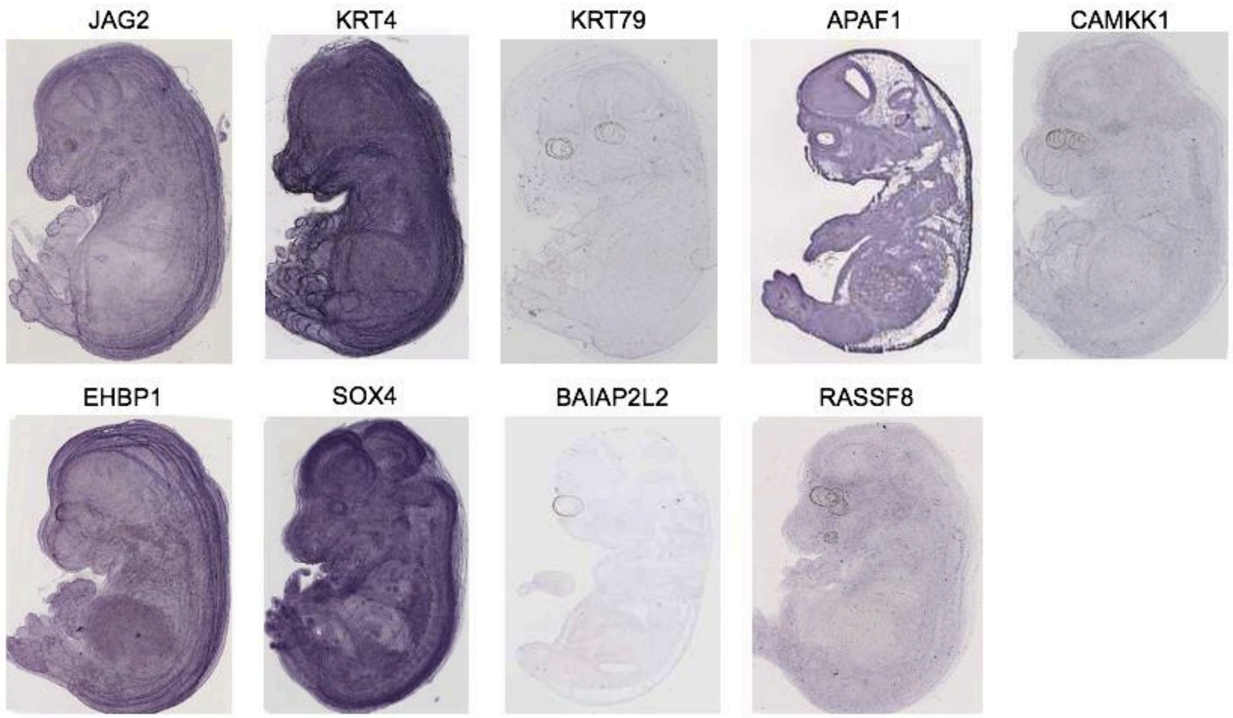

Fig S2. *In situ* hybridization of additional gene hits in 14.5 embryonic mice. Genes already available on the eMouseAtlas exhibit varying levels of expression in the brain during this stage of development, with *Baiap2l2* and *Krt79* showing minimal expression. Image credit: EMAGE Project (EMAGE: 3758, 16846, 14151, 12754, 14122, 12668, 13766, 29351, 11832).

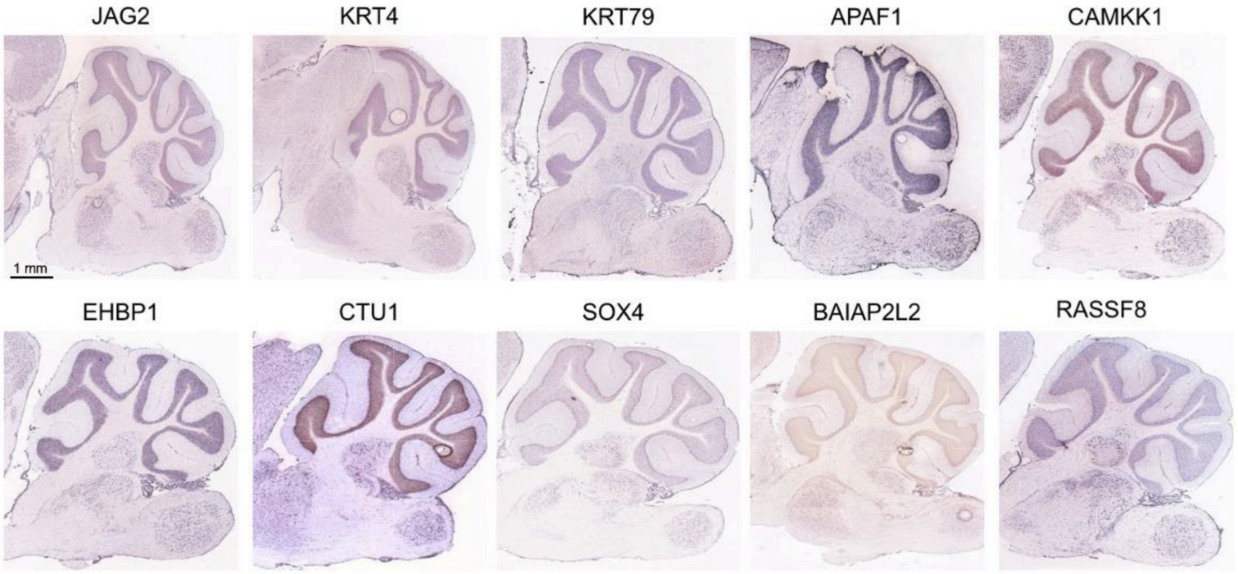

Fig S3. *In situ* hybridization of additional gene hits in adult mice. All genes already recorded by the Allen Brain Atlas show some level of mRNA expression, with *Camkk1*, *Ctu1*, and *Rassf8* showing the most elevated levels across samples. Scaling is equal across images. Image credit: Allen Institute (https://mouse.brain-map.org/experiment/show?id=69735652, https://mouse.brain-map.org/experiment/show?id=69735956, https://mouse.brain-map.org/experiment/show?id=68522308, https://mouse.brain-map.org/experiment/show?id=68745275, https://mouse.brain-map.org/experiment/show?id=70431211, https://mouse.brain-map.org/experiment/show?id=70239099, https://mouse.brain-map.org/experiment/show?id=413826, https://mouse.brain-map.org/experiment/show?id=81654151, https://mouse.brain-map.org/experiment/show?id=74431104, https://mouse.brain-map.org/experiment/show?id=69443280).
